# Effects of Materialism on Adolescents’ Prosocial and Aggressive Behaviors: The Mediating Role of Empathy

**DOI:** 10.3390/bs13100863

**Published:** 2023-10-21

**Authors:** Meijing Lv, Mengyuan Zhang, Nianhui Huang, Xinyuan Fu

**Affiliations:** School of Sociology and Psychology, Central University of Finance and Economics, Beijing 100081, China

**Keywords:** materialism, prosocial behavior, aggressive behavior, empathy, dehumanization

## Abstract

Materialism plays a critical role in adolescent behavioral development, yet whether it affects prosocial and aggressive behaviors and the internal mechanism remains unknown. Therefore, this longitudinal research examined the relationships between adolescent materialism and prosocial and aggressive behaviors, and tested the mediating effect of empathy. In 2015, we recruited 543 adolescents from four junior high schools in Beijing, China (284 girls, 259 boys; *M* = 11.27 years, *SD* = 0.51). The participants completed the measures of materialism and demographic information at the initial time point, completed the measure of empathy about one year later, and completed the measures of prosocial and aggressive behaviors after about another year. The hypotheses were tested using a structural model using maximum likelihood estimation. The mediating effects were estimated by taking 1000 bias-corrected bootstraps. The results revealed that materialism was associated with aggressive behavior directly and positively, but had no significant correlation with prosocial behavior. Materialism had an indirect and negative correlation with prosocial behavior via empathy, while no indirect effect of materialism on aggressive behavior was found. The findings add to our knowledge of the dehumanizing nature of materialism by revealing its effect on adolescent behavioral development, as well as the underlying mechanism.

## 1. Introduction

Materialism refers to a suite of goals and values that focus upon wealth, possessions, image, and status [1]. It is a system of fundamental human values/goals that are in conflict with goals related to the well-being of others and one’s own growth [2]. Materialism affects people in a negative way. According to convincing evidence, adults who give high priority to materialistic values/goals consume more products and take on more debt, have lower-quality interpersonal relationships, behave in more ecologically detrimental ways, and report less physical and psychological well-being [3,4,5]. However, comparatively speaking, regarding adolescent materialism, the existing literature focuses relatively more on its causes than on its consequences.

With respect to the causes, adolescent materialism is mainly shaped via two pathways: via the experiences that elicit insecurity feelings and via exposure to the social models that encourage materialistic goals/values [6]. With regard to consequences, previous research has shown that adolescent materialism is predictive of increased risky behavior engagement [7], more delinquent behavior [8], lower generosity [9], and so on. Research has highlighted such behavioral consequences, yet whether adolescent materialism affects other kinds of behaviors remains unknown. Considering that both prosocial and aggressive behaviors are crucial aspects of adolescent behavioral development, the current study looks into whether adolescent materialism is related to prosocial and aggressive behaviors and why, via examining the mediating role of empathy. We believe that answering these research questions will help to understand the relationship between adolescent materialism and behavioral development and its underlying mechanism, as well as help to develop educational programs and intervention projects that promote the positive development of materialistic adolescents. 

### 1.1. Roles of Materialism on Prosocial and Aggressive Behaviors

Both theoretical and empirical evidence suggests that values guide individual behavior [1,2]. As a value, materialism plays a destructive role in the positive development of adolescents, such as lower generosity [9], lower subjective well-being [10], lower school well-being [11], and reduced consumer ethical beliefs [12]. Studies have also found that materialism has positive associations with psychological and behavioral problems in adolescents, such as increased risky behavior engagement [7], more body shame [13], and problematic smartphone use [14]. To follow this line of research further, we opted to test whether materialism has a negative correlation with prosocial behavior and a positive correlation with aggressive behavior among adolescents.

According to what we have learned, there are two empirical studies that have focused on the relation between materialism and prosocial behavior. One study, based on adult samples, revealed that materialism altered the impact of accounting for time on prosocial behavior; that is, accounting for time decreased the time and money spent on others for participants holding moderate levels of materialism, but not for the participants with low or high levels of materialism [15]. The other study found that adolescent materialism was longitudinally associated with decreased prosocial behavior toward friends and strangers, but not toward the family [16]. Moreover, only a handful of research has provided hints on the influence of materialism on aggressive behavior. For example, an Israeli study has shown that materialism is a trigger of road aggressiveness [17]. According to a study based on a sample of Chinese adolescents, materialism positively predicted delinquent behavior [8]. Hence, whether materialism is predictive of adolescent aggressive behavior remains out of focus. Given such evidence of associations between materialism and adolescent behavioral outcomes, and that values guide individual behavior [1,2], it is reasonable to speculate that materialism has a negative association with adolescents’ prosocial behavior and a positive association with aggressive behavior.

### 1.2. Mediating Role of Empathy

More and more studies have focused on the impact of materialism in adolescent psychological and behavioral development; however, there is an important knowledge gap regarding the internal mechanism. Because materialism can promote a culture of dehumanization where people lack empathy for others [18], and empathy plays a crucial role in prosocial and aggressive behaviors [19], we propose that empathy might be a potential mediator of the associations between adolescent materialism and prosocial and aggressive behaviors.

Theoretically, the dehumanizing perspective of materialism claims that materialists value material possessions over human connections and relationships, leading to a tendency to view other people as objects rather than individuals with their own thoughts, feelings, and experiences [20]. This dehumanization can lead to a situation where materialists become disconnected from the needs and concerns of others and, hence, lack empathy for others [21]. Furthermore, empathy is a key motivator for engaging in prosocial behavior and relates inversely to aggressive behavior [22,23]. According to the empathy-altruism hypothesis [19], when people feel more empathy for others, they are more likely to understand and share the feelings of others, and to respond to them in a caring and compassionate way [24]. Thus, they are more likely to implement prosocial behavior. Besides, individuals with higher empathy are more likely to be able to regulate their emotions and impulses, and to recognize the impacts of their behaviors on others [25]. As a result, they are less likely to engage in aggressive behavior, which can cause harm or distress to others. In sum, it is theoretically logical to assume empathy as a mediator in the links between adolescent materialism and prosocial and aggressive behaviors.

Empirically, research has found that adolescent materialism is detrimental to empathy, and empathy is predictive of prosocial and aggressive behaviors. For instance, an investigation of Hong Kong adolescents revealed a negative relationship between materialism and empathy [26]. A longitudinal study showed that adolescent sympathy had a positive link with prosocial behavior a year later [27]. A study regarding a cross-sectional sample of Polish adolescents found that high affective empathy predicted decreased bullying perpetration [28]. Combining the above theoretical and empirical evidence, there is a significant correlation between adolescent materialism, empathy, prosocial behavior, and aggressive behavior, and we speculate that empathy mediates the relationships between adolescent materialism and prosocial and aggressive behaviors.

### 1.3. Overview of the Present Research

This research investigates the relationships between adolescents’ materialism and prosocial and aggressive behaviors, and tests the mediating effect of empathy. Based on the theoretical claim that values guide individual behavior and the dehumanizing perspective of materialism, as well as the empirical literature regarding the detrimental impact of materialism on adolescent behavioral development [7,8,9], we formulated the hypotheses of the study. We hypothesized that materialism would be associated with prosocial behavior negatively (Hypothesis 1) and be associated with aggressive behavior positively (Hypothesis 2). We further hypothesized that materialism would correlate with empathy negatively, which, in turn, would be positively correlated with prosocial behavior (Hypothesis 3); materialism would correlate with empathy negatively, which, in turn, would be negatively correlated with aggressive behavior (Hypothesis 4). To answer the research questions, we used a longitudinal design with three waves of data over three years to test the above hypotheses by recruiting a sample of Chinese adolescents.

## 2. Method

### 2.1. Participants

The participants were recruited from four junior high schools in Fangshan District, Beijing, China. The first-year students in these junior high schools were sixth-graders, due to the five–four educational system (i.e., five years for primary school and four years for junior high school). In total, 543 first-year students were recruited at Time 1 in 2015, including 284 girls and 259 boys (*M*_age in 2015_ = 11.27 years, *SD*_age in 2015_ = 0.51). About one year later, 449 of them participated at Time 2 in 2016. Ninety-four participants were unable to attend due to commitments, leading to a 17.31% attrition rate. After about another year, 417 adolescents took part at Time 3 in 2017. Because 8 of the 417 participants missed the survey at Time 2, there were 409 participants who participated in all three waves of the survey.

### 2.2. Procedure

The study was approved by the Institutional Review Board of the Central University of Finance and Economics. Convenience sampling was used. We invited the four schools via personal social networking, and they all agreed to participate in our survey. We informed the target schools’ headmasters of our study’s goal and obtained their consent before data collection. The teachers at each school introduced the survey to all first-year students, and explained that it was about their psychological development and the data would be kept confidential. Those students who agreed to participate in all three rounds of the survey over the three years offered written informed consent from both their parents and themselves. At each time point, the survey took about 15 min; the participants were tested in groups in quiet classrooms with a research assistant present who collected data and kept order in the classroom. When the participants began filling in the questionnaire, the research assistant stayed at the front of the classroom, keeping a distance from the students to prevent social desirability bias. Finally, the participants were debriefed and given notebooks after completing the survey (i.e., notebooks, pencil sharpeners, or pens) in appreciation of their participation. To ensure the accurate translation of the scales (i.e., materialism, empathy, prosocial behavior, and aggressive behavior), which were originally in English, two graduates of English were hired to translate and back-translate them [29].

Regarding the above survey, the omnibus (or multi-thematic) panel design was used, in which the same participants were measured on more than one occasion, but the variables measured varied from one time point to another [30]. The variables in this omnibus survey contained the measures of our study and the schools’ psychological development census (e.g., depression, anxiety, parent–child relationship, and personality). Accordingly, materialism and demographic information were measured in the first year, empathy was assessed one year later, and prosocial and aggressive behaviors were measured in the third year. Measuring the predictor, mediator, and outcome variables, respectively, at three time points rather than at one time point could reduce common method biases and, thus, strengthen the robustness of the results [30]. All of the measures were appropriate for use in adolescents [31,32,33,34]. It should be noted that our research was the primary study with exclusive purposes rather than a secondary data analysis of the schools’ psychological development census, whose objective was different. In addition, we utilized student numbers as the identifiers to merge data from the three time points.

### 2.3. Measures

#### 2.3.1. Materialism

Materialism was measured at Time 1 via six items adapted from the Youth Materialism Scale [31]. The original scale contained 10 items, but four of them were excluded due to their cultural inapplicability. For instance, the excluded item “I’d rather not share my snacks with others if it means I’ll have less for myself” may measure generosity rather than materialism in the Chinese cultural context. It was a six-point Likert-type scale from 1 (totally disagree) to 6 (totally agree). Sample items included “When you grow up, the more money you have, the happier you are”. Higher average scores indicated greater materialism.

#### 2.3.2. Empathy

The empathy of adolescents at Time 2 was assessed by a seven-item measure [32]. Each item was rated on a five-point Likert-type scale from 1 (strongly disagree) to 5 (strongly agree). After reverse coding two of the items, higher average values indicated higher levels of empathy. Sample items included “I am often quite touched by things that I see happen” and “when I see someone being taken advantage of, I feel kind of protective towards them”.

#### 2.3.3. Prosocial Behavior

We measured adolescent prosocial behavior using a revised version of the kindness and generosity subscale within the Values in Action Inventory of Strengths [33] at Time 3. The original scale contained nine items, and two of them were excluded due to the fact that their factor loadings were lower than 0.40. The participants rated each item (e.g., “I go out of my way to cheer up people who seem sad, even if I do not know them”) on a five-point Likert-type scale (1 = very much unlike me, 5 = very much like me). Higher average values meant more prosocial behavior.

#### 2.3.4. Aggressive Behavior

At Time 3, we measured aggressive behavior using the five items from Weinberger and Schwartz [34]. Adolescents reported on a five-point Likert-type scale (1 = does not describe me, 5 = describes me very well). Sample items included “I pick on people I don’t like” and “people who get me angry better watch out”. Higher average values indicated higher levels of aggressive behavior.

### 2.4. Data Analyses

First of all, the missing data and attrition analyses were performed to examine whether there were any differences between the 409 participants who stayed in the study and the 134 participants who dropped out at Time 2 and/or 3. Secondly, we used SPSS (version 26) to perform descriptive statistics and correlated analysis for the key variables. Thirdly, confirmatory factor analysis was performed for each latent construct using Mplus (version 8.3). In the fourth step, the research hypotheses were tested using a structural model with maximum likelihood estimation. Finally, we estimated the mediating effects by taking 1000 bias-corrected bootstraps. It should be noted that full information maximum likelihood (FIML), which is a missing data estimation and imputation approach for structural equation modeling, was used in the present study. Therefore, the analytic sample included 543 participants.

## 3. Results

### 3.1. Characteristics of the Sample

In the analytic sample (*n* = 543), 22.10% of fathers and 25.60% of mothers had a junior high school diploma or lower, 33.15% of fathers and 31.31% of mothers possessed a high school diploma or equivalent educational level, 16.21% of fathers and 20.07% of mothers had a university degree, 17.50% of fathers and 14.73% of mothers had a bachelor’s degree, 3.31% of fathers and 2.21% of mothers had a master’s degree or higher, and 7.73% of fathers’ and 6.08% of mothers’ data were missing. Regarding subjective family economic status, 0.55% perceived their family as extremely poor, 4.97% perceived their family as poor, 70.17% perceived their family as average, 17.86% perceived their family as rich, 0.37% perceived their family as extremely rich, and 6.08% of the data were missing.

### 3.2. Missing Data and Attrition Analyses

For the study sample (*n* = 543), the missing values of the variables were from 0% to 23.76%, resulting in a total of 14.30% missing data. Little’s Missing Completely at Random (MCAR) Test was performed, and the result indicated our data were missing completely at random, χ^2^ (864) = 819.45, *p* = 0.859. Subsequently, *t*-tests and a Chi-square test were executed, showing that those 409 maintained participants were different from the 134 participants who dropped out at Time 2 and/or 3. The results indicated that participants with older age (*p* = 0.023), whose parents had lower levels of education (fathers: *p* < 0.001; mothers: *p* < 0.001), or with lower subjective family economic status (*p* = 0.001) were more prone to failure in providing responses at Time 2 and/or 3. No significant differences were found between these two groups regarding gender (*p* = 0.311) and materialism (*p* = 0.381). In summary, the sample became younger and became more biased toward better-educated and higher-economic-status families, but did not experience attrition regarding gender and materialism.

### 3.3. Reliability and Validity of the Measures

For materialism, Cronbach’s alpha was 0.80 in the present study. A latent variable was created for it. The model fit of confirmatory factor analysis with modifications was acceptable: χ^2^ (3) = 8.37, *p* = 0.039, CFI = 0.995, TLI = 0.975, RMSEA = 0.057, SRMR = 0.014. The factor loadings varied from 0.56 to 0.76. For empathy, the items formed a latent variable (Cronbach’s alpha = 0.75). The confirmatory factor analysis with modifications showed a good model fit: χ^2^ (9) = 19.18, *p* = 0.024, CFI = 0.985, TLI = 0.966, RMSEA = 0.050, SRMR = 0.028, and the factor loadings ranged from 0.41 to 0.74. For prosocial behavior, Cronbach’s alpha was 0.92 in the present study. We created a latent variable for it. The confirmatory factor analysis with modifications indicated good model fit: χ^2^ (10) = 14.60, *p* = 0.147, CFI = 0.998, TLI = 0.995, RMSEA = 0.033, SRMR = 0.016. The factor loadings varied from 0.69 to 0.85. For aggressive behavior, we created a latent variable for it (Cronbach’s alpha = 0.84). The model fit of confirmatory factor analysis with modifications was good: χ^2^ (3) = 6.16, *p* = 0.104, CFI = 0.997, TLI = 0.989, RMSEA = 0.050, SRMR = 0.016, with factor loadings ranging from 0.41 to 0.89.

### 3.4. Descriptive Statistics and Correlations

Table 1 displays the variables’ descriptive statistics and correlations. The result showed that adolescents’ materialism at Time 1 had a negative correlation with empathy at Time 2, and a positive correlation with aggressive behavior at Time 3. Empathy at Time 2 was positively related to prosocial behavior at Time 3, and was negatively correlated with aggressive behavior at Time 3. Prosocial behavior at Time 3 was negatively correlated with aggressive behavior at Time 3.

### 3.5. Structural Model

In this structural model, we included materialism at Time 1 as the predicting variable, empathy at Time 2 as the mediator, prosocial and aggressive behaviors at Time 3 as the outcome variables, and controlled for gender, age, and subjective family economic status at Time 1. The normality of the observed variables was tested via skewness and kurtosis. The results showed that the skew values were between −1.196 and 2.055, and the kurtosis values were between −1.992 and 5.129. It has been argued that data are considered to be normal if the skewness is between −2 and +2 and the kurtosis is between −7 and +7 [35,36]. Thus, the structural model met the normality assumption, except for one item with a skew value (equal to 2.055) slightly larger than 2. The final analytic sample for the structural model included 504 participants due to missing data on predictor variables in 39 cases. Regarding the adequacy of the sample size for the mediation analysis, the medium effect size (*f*^2^ = 0.15) suggested that at least 138 participants were needed to obtain 95% power with an α of 0.05 (power analysis was performed in G*Power version 3.1.9.6). Hence, the present sample size was adequate.

**Figure 1:**
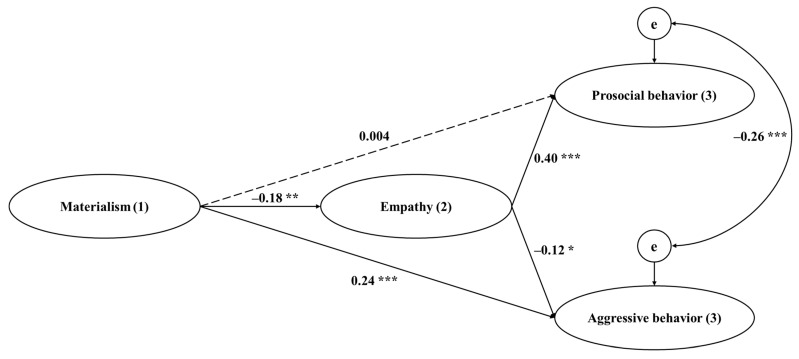
Effects of materialism on prosocial and aggressive behaviors via empathy. Note: Standardized coefficients are presented. The dashed line represents the non-significant path. Control variables are omitted for parsimony. χ^2^ (321) = 546.77, *p* < 0.001, CFI = 0.951, TLI = 0.943, RMSEA = 0.037, SRMR = 0.051. (1) = Time 1; (2) = Time 2; (3) = Time 3. * *p* < 0.05, ** *p* < 0.01, *** *p* < 0.001.

The final model fit the data well: χ^2^ (321) = 546.77, *p* < 0.001, CFI = 0.951, TLI = 0.943, RMSEA = 0.037, SRMR = 0.051. As indicated in Figure 1, the direct effect of materialism was not significant for prosocial behavior (β = 0.004, *p* = 0.941, 95% CI = [−0.106, 0.115]), but was significant for aggressive behavior (β = 0.24, *p* < 0.001, 95% CI = [0.134, 0.353]). Hypothesis 1 was not supported, whereas Hypothesis 2 was verified. Moreover, participants’ materialism had a negative correlation with empathy (β = −0.18, *p* = 0.002, 95% CI = [−0.298, −0.064]), which then positively associated with prosocial behavior (β = 0.40, *p* < 0.001, 95% CI = [0.289, 0.504]) and negatively associated with aggressive behavior (β = −0.12, *p* = 0.046, 95% CI = [−0.236, −0.002]). There was a significant negative correlation between prosocial behavior and aggressive behavior (β = −0.26, *p* < 0.001, 95% CI = [−0.370, −0.153]). Additionally, the indirect effect of materialism on prosocial behavior via empathy was significant (β = −0.07, *p* = 0.013, 95% CI = [−0.135, −0.022]). Hypothesis 3 was verified. However, the indirect path from materialism to aggressive behavior via empathy was not significant (β = 0.02, *p* = 0.144, 95% CI = [−0.001, 0.055]). Hypothesis 4 was not supported. Regarding the control variables, girls showed less aggressive behavior than boys did (β = 0.14, *p* = 0.004, 95% CI = [0.046, 0.241]). In terms of age, it had a positive correlation with aggressive behavior (β = 0.12, *p* = 0.022, 95% CI = [0.018, 0.227]). Subjective family economic status had a positive correlation with prosocial behavior (β = 0.12, *p* = 0.015, 95% CI = [0.023, 0.216]). No other significant effects of the control variables were found.

## 4. Discussion

### 4.1. Effects of Adolescent Materialism on Prosocial and Aggressive Behaviors

According to the results, the direct effect of adolescent materialism was not significant for prosocial behavior, but was significant for aggressive behavior. Prosocial behavior and aggressive behavior were negatively correlated with each other, which is consistent with previous research [37,38]. The positive relationship between adolescent materialism and aggressive behavior could be explained by the following two reasons. On the one hand, materialists generally focus on external goals such as wealth and status, which makes them more self-focused and competition-oriented [8]. This self-centralization and competition orientation might cause indifference and tension in interpersonal relationships, and can result in interpersonal hostility and conflict, hence leading to more aggressive behaviors among materialistic adolescents [39]. On the other hand, due to the constant pursuit of material goals, materialists can easily fall into an emotional state of stress, anxiety, and frustration once they fail. This emotional state could be a trigger for aggressive behavior according to the frustration–aggression hypothesis [40]. Accordingly, materialistic adolescents are more likely to engage in aggressive behaviors than their counterparts.

Our study did not find a direct effect of adolescent materialism on prosocial behavior. This may be due to the double-edged nature of the direct path. On the one hand, implementing prosocial behaviors brings benefits to materialistic adolescents, such as higher status among peers [41] and better relationships with others, especially with those who have abundant material and social resources [42]. Because both of the above are highly valued by materialists [2], those adolescents with higher materialism may perform more prosocial behaviors, though more likely for self-interest than for altruistic purposes. On the other hand, due to the dehumanizing nature of materialism, materialistic adolescents may become disconnected from the needs and concerns of others and, hence, perform fewer prosocial behaviors [21]. To sum up, these two contradictory effects might counteract each other and result in the failure of materialism to directly predict adolescent prosocial behavior.

### 4.2. Mediating Effects of Empathy

As hypothesized, the indirect effect of materialism on prosocial behavior via empathy was significant. This is consistent with previous research revealing that adolescent materialism was negatively related to empathy [26], and that empathy was positively predictive of prosocial behavior [27]. The process of internalizing materialistic values prompts adolescents to dehumanize others. That is, materialistic adolescents usually prioritize their own wants and needs over those of others, and focus on how to take advantage of others to actualize their own material pursuits [8]. This way of treating others makes these materialistic teenagers less concerned about the pain and hardships others go through, gradually resulting in low empathy. In other words, adolescent materialism restrains the development of empathy. According to the empathy-altruism hypothesis, empathy is the core antecedent of prosocial behavior [19]. Specifically, those individuals feeling high levels of empathy for others in need will be more likely to help than will those feeling less empathy [43]. Similarly, those materialistic adolescents feeling low levels of empathy are less motivated to help than their counterparts. In other words, lacking empathy leads to insufficient prosocial acts. Hence, adolescent higher materialism is linked to lower empathy, which, in turn, leads to less prosocial behavior, as found in the present study.

However, the indirect path from adolescent materialism to aggressive behavior via empathy was not significant. Though the path from materialism to empathy was significant, as discussed earlier, the path from empathy to aggressive behavior was not significant. This is inconsistent with previous research suggesting a negative relationship between empathy and aggression in adolescents [44]. As a decrease in empathy implies more indifference to the claims of others, individuals with poor empathy are often perceived by others as having a cold and indifferent personality [45]. But at the same time, a lack of empathy does not imply actively attacking others. There is empirical evidence showing that empathy is not predictive of aggressive behavior [46]. Hence, reduced empathy due to materialism may not, in itself, be enough to trigger aggressive behavior, as the latter is more often triggered by convergent motives such as frustration and revenge [47]. Therefore, the mediating effect of empathy on the relationship between adolescent materialism and aggressive behavior was non-significant.

### 4.3. Limitations and Future Directions

There are some deficiencies in this study. First of all, though the study variables were assessed over a three-year period, it was an observational study and initial levels of the mediator and outcome variables were not measured and controlled for due to the omnibus (or multi-thematic) panel design of the survey. Thus, caution is needed when making causal inferences about the results. It would be beneficial to include both of the above in future research. Second, our study focused on a Chinese sample. Chinese culture emphasizes expressive suppression, which may discourage adolescents from disclosing materialistic values, hence leading to an underestimation of the relationships between the variables. Thus, the findings may be tied to this specific culture and cannot be generalized to others. It is desirable to replicate our research in different cultures. Third, convenience sampling was used and the size was relatively small, which might weaken the robustness of the results. A more rigorous sampling strategy and larger sample size would be desirable in future work. Fourth, the attrition in our sample regarding age, parental educational level, and subjective family economic status might confound the studied effects, though no attrition was found regarding gender and materialism. Therefore, more complete longitudinal samples would be fruitful to study the effects. Fifth, though empathy was found to have a mediating role regarding the relationship between adolescent materialism and prosocial behavior, there might be other potential mediators, such as self-regulation and self-esteem. Therefore, it is necessary to identify these possible underlying mechanisms in future studies.

## 5. Conclusions

This longitudinal study investigated the relationships between materialism and prosocial and aggressive behaviors among adolescents. In addition, we tested the mediating effect of empathy regarding the above relationships. With three waves of data collected over three years in China, it was found that materialism was directly and positively related to aggressive behavior, but it had no direct correlation with prosocial behavior. Moreover, materialism had an indirect and negative correlation with prosocial behavior via empathy, while no indirect effect of materialism on aggressive behavior was found. Overall, the findings add to our knowledge of the dehumanizing nature of materialism by revealing its effect on adolescent behavioral development, as well as the underlying mechanism.

The present study makes certain contributions. At the theoretical level, this study has deepened our understanding of the dehumanizing perspective of materialism and that values guide individual behavior. At the empirical level, it helps to better understand the relationship between adolescent materialism and behavioral development via focusing on both prosocial and aggressive behaviors, as well as the underlying mechanism in terms of empathy. At the practical level, given the effects of materialism on adolescent prosocial and aggressive behaviors, establishing and maintaining a healthy school climate that values interpersonal connections and relationships over material possessions could be an effective strategy for promoting prosocial behavior and reducing aggressive behavior among adolescents. In addition, educational programs and intervention projects that optimize empathy may boost the positive development of materialistic adolescents.

## Figures and Tables

**Table 1:** Descriptive statistics and correlations for the key variables.

Variable	1	2	3	4
1. Materialism at Time 1				
2. Empathy at Time 2	−0.14 **			
3. Prosocial behavior at Time 3	−0.08	0.31 ***		
4. Aggressive behavior at Time 3	0.20 ***	−0.16 **	−0.24 ***	
*M*	2.10	3.89	3.63	1.81
*S.D.*	1.08	0.63	0.88	0.73

Note: The analytic sample for Time 1 consisted of 543 participants, for Time 2 consisted of 449 participants, and for Time 3 consisted of 417 participants. ** *p* < 0.01, *** *p* < 0.001.

## Data Availability

The data presented in this study and all Mplus syntax files are openly available at OSF accessed on 20 August 2023: https://osf.io/u72fy/?view_only=8742fca1a94148a7853b51ab99ee0dbf.
